# Supplementary material for: In-Depth Characterization of a Re-Engineered Cholera Toxin Manufacturing Process Using Growth-Decoupled Production in Escherichia coli
Source: Toxins (Basel). 2022 Jun 8;14(6):396. doi: 10.3390/toxins14060396 (PMC9228256; doi:10.3390/toxins14060396)
Supplement: Supplementary file 1 [file toxins-14-00396-s001.zip › toxins-1726057-supplementary.pdf]

In-Depth Characterization of a Re-Engineered Cholera Toxin Manufacturing Process Using Growth-Decoupled Production in *Escherichia Coli*

Natalia Danielewicz, Wenyue Dai, Francesca Rosato, Michael E. Webb, Gerald Striedner, Winfried Römer, W. Bruce Turnbull and Juergen Mairhofer

(a)

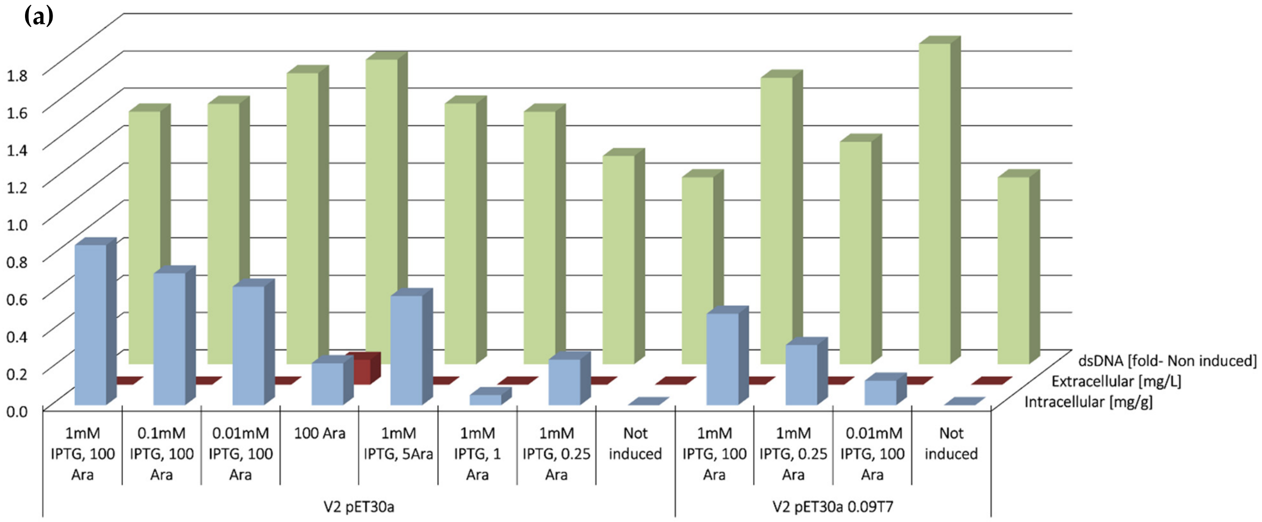

(b)

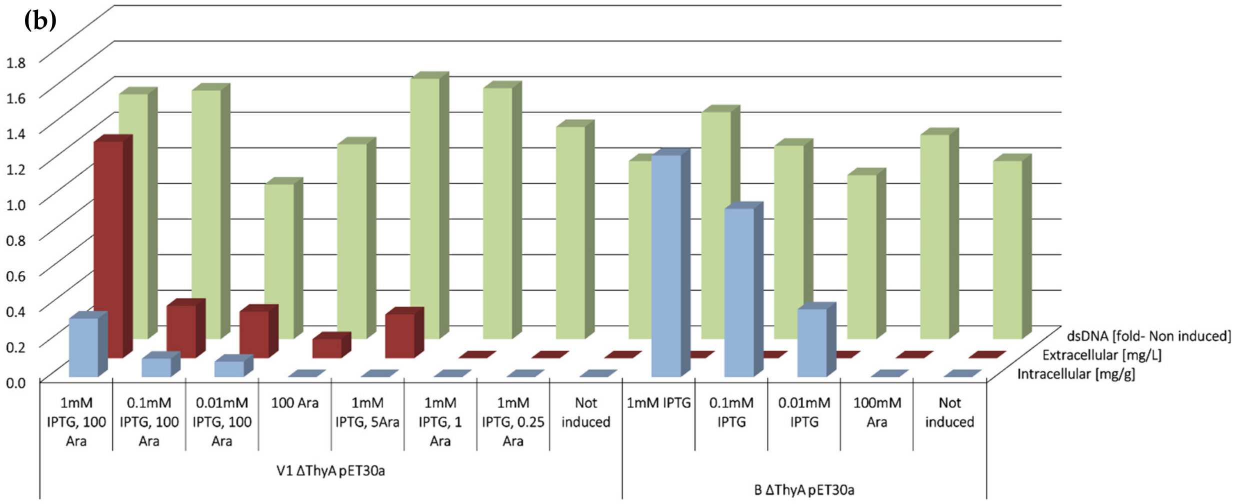

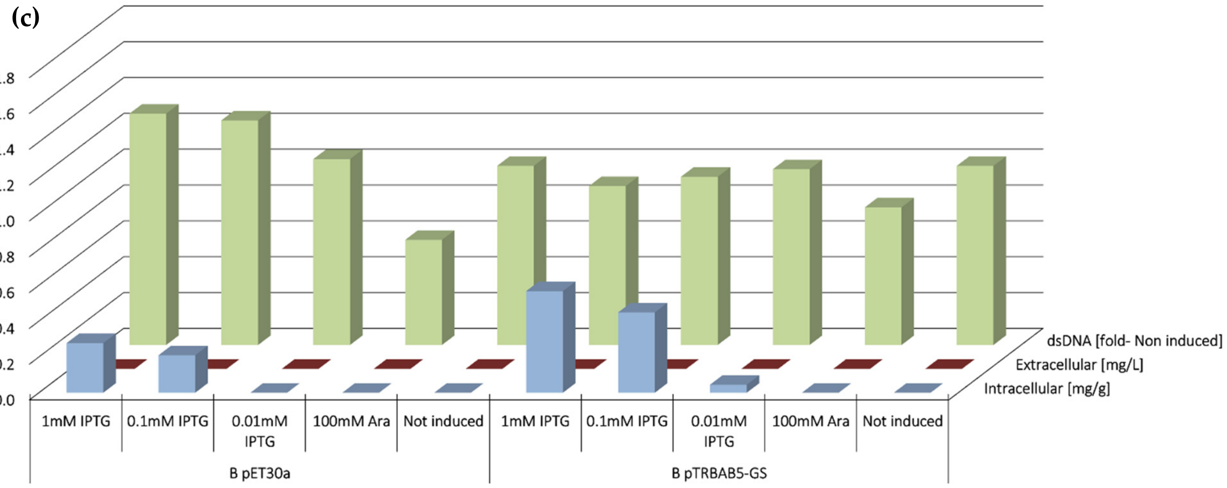

Figure S1. Summary of micro-scale fermentations performed with (**a**) V2, (**b**) V1/B *ΔthyA* and (**c**) B strains induced with different concentrations of IPTG (1-0.01mM) and Ara (100-0.24mM).

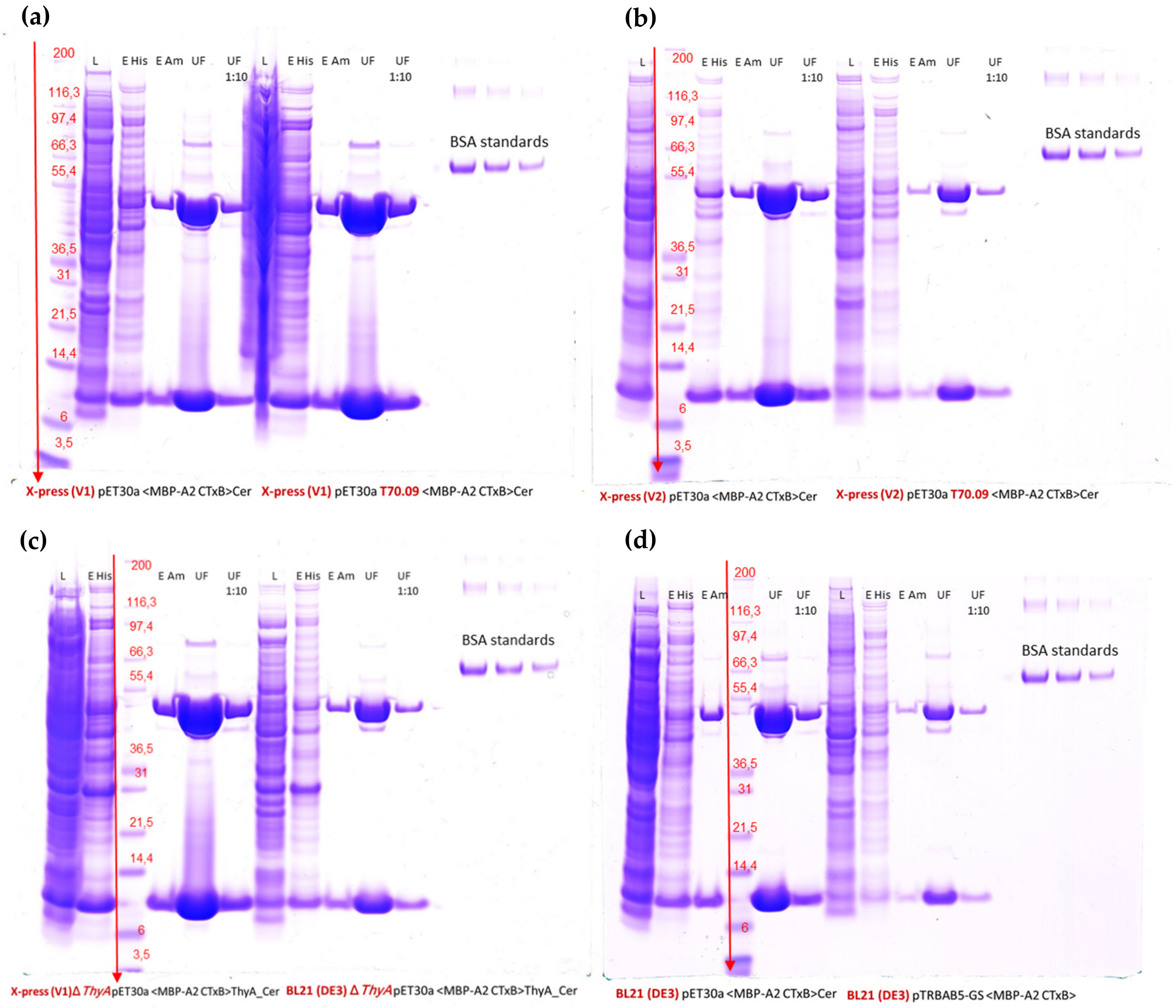

Figure S2. SDS-PAGE gel with fractions of rCTC purification and formulation. (**a**) V1 pET30a <MBP-A2 CTxB>Cer and V1 pET30a T70.09 <MBP-A2 CTxB>Cer (**b**) V2 pET30a <MBP-A2 CTxB>Cer and V2 pET30a T70.09 <MBP-A2 CTxB>Cer (**c**) V1 *ΔthyA* pET30a <MBP-A2 CTxB>ThyA_Cer and BL21 (DE3) *ΔthyA* pET30a <MBP-A2 CTxB>ThyA_Cer (**d**) BL21 (DE3) pET30a <MBP-A2 CTxB>Cer and BL21 (DE3) pTRBAB5-G1S <MBP-A2 CTxB>. L-loading, E-elution, His-Ni^{+2} His-Trap FF, Am-Amylose High Flow and UF-ultrafiltration.

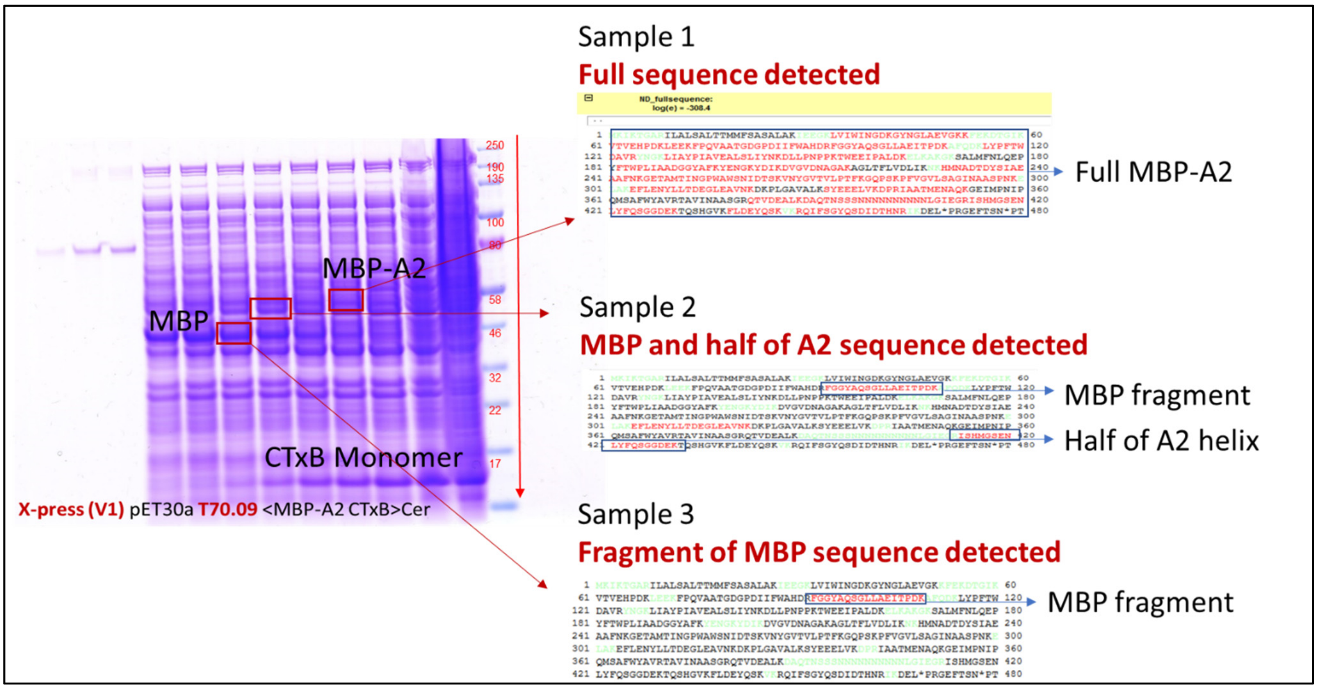

Figure S3. LC-ESI-MS/MS peptide mapping MS analysis of selected bands from extracellular soluble SDS-PAGE gel.

(a)

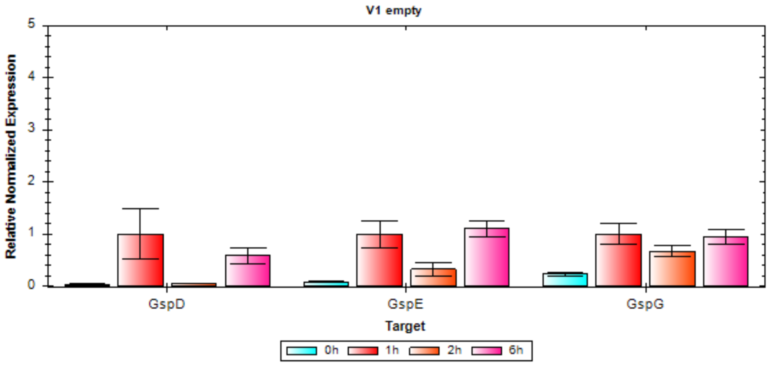

(b)

SigGenes logFC (post induction)

GeneID	1h	3h	7h	13h
gspA	1.31		1.81	
gspB	0.84		1.15	
gspC			1.06	1.55
gspD	1.11		1.73	1.08
gspE	1.22	1.17	1.91	1.01
gspF	1.28	0.99	2.16	
gspG	1.56	1.22	2.42	
gspH	1.62		2.24	
gspI	1.50		1.88	
gspJ	1.44		2.14	
gspK			1.69	
gspL	1.60	1.21	2.06	
gspM	0.91		1.49	
gspO			0.92	
hns				-1.25

Figure S4. Analysis of V1 empty strain using (**a**) qPCR screen of *gspD*, *gspE*, *gspG* expression analysed against reference *cysG* and *rssA* housekeeping genes, and (**b**) RNA full genome transcriptomics data.

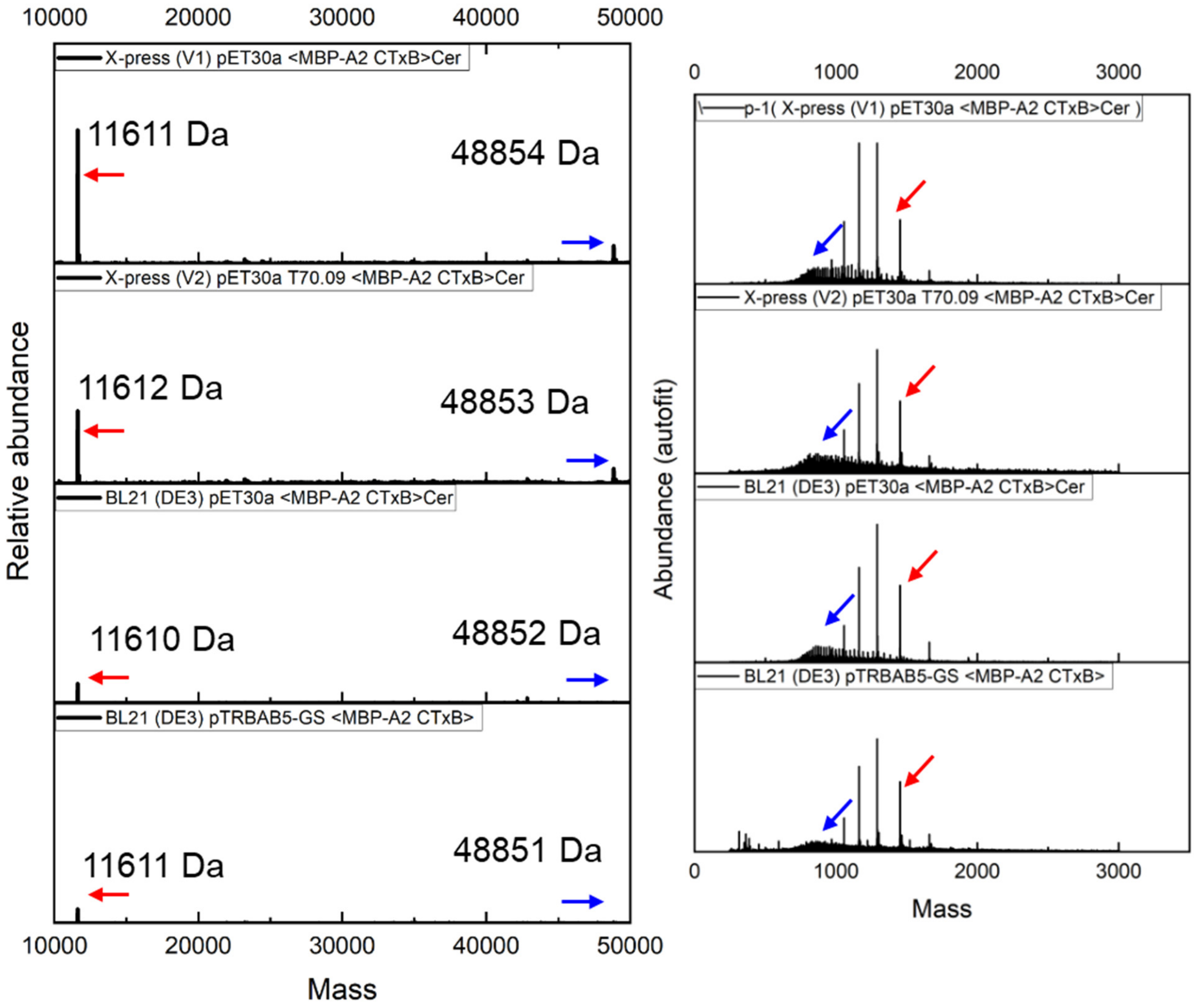

Figure S5. MS analysis of formulated rCTC.

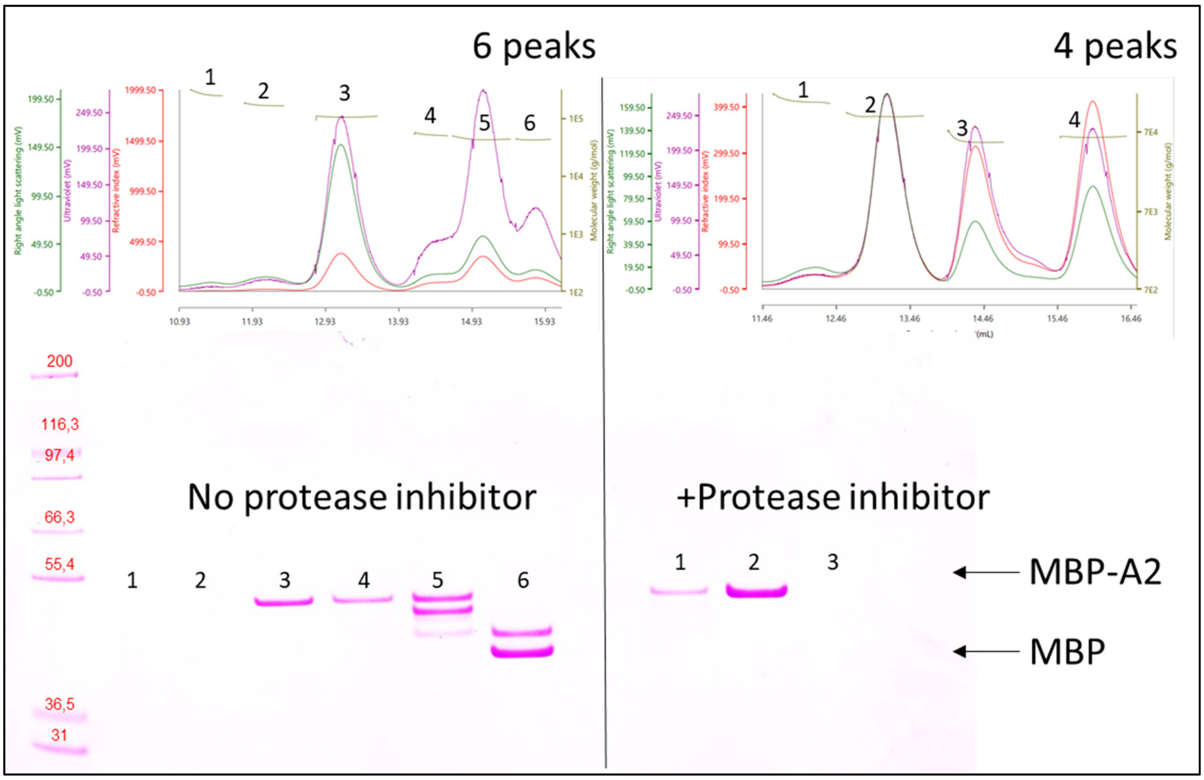

Figure S6. Influence of protease inhibitor on purified rCTC. Presence of MBP-A2 and digested MBP confirmed with MS (data not included).

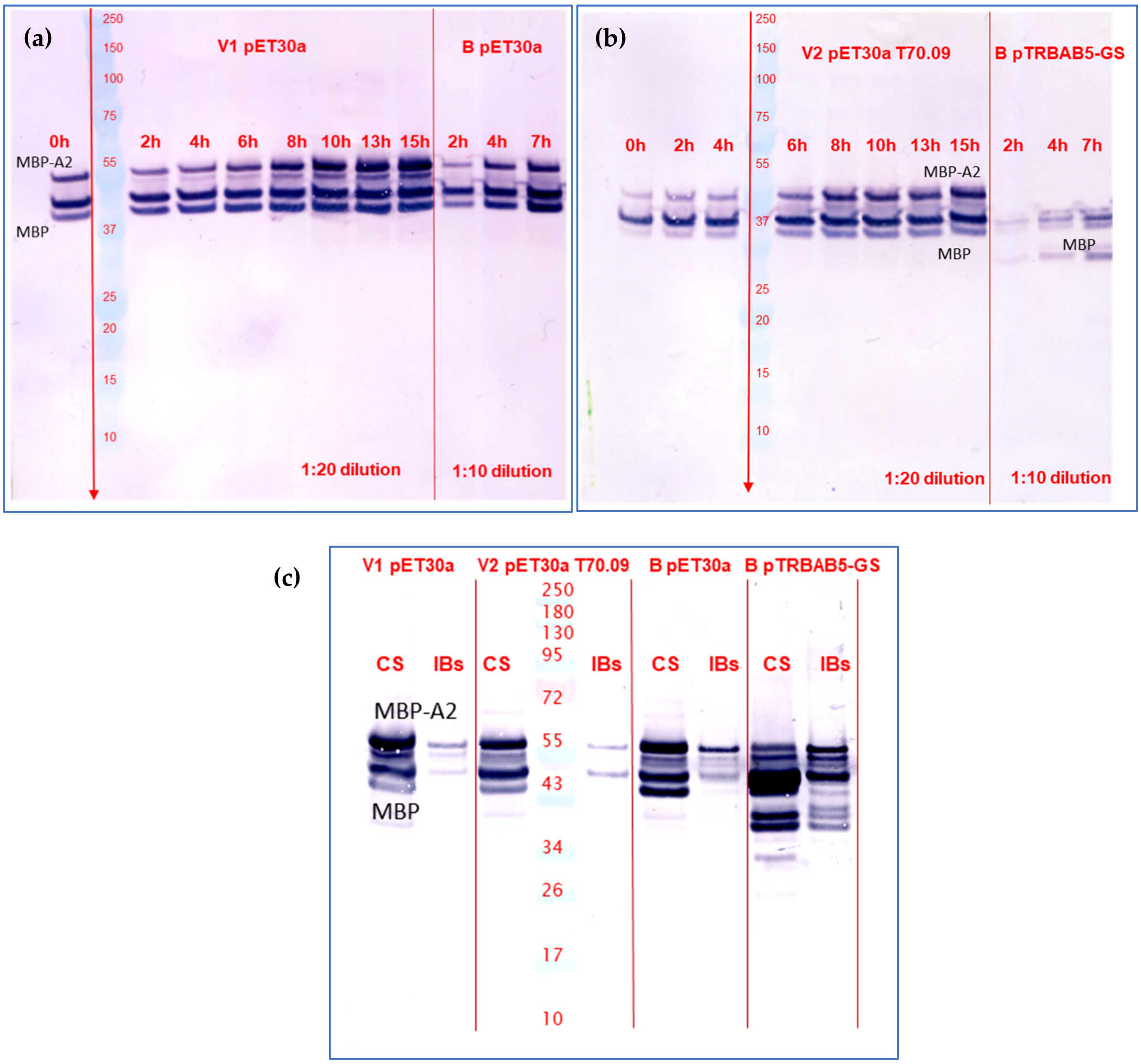

Figure S7. Anti-MBP Western Blot of MBP-A2 and its by-product produced extracellular and intracellular. (**a**) V1 pET30a<MBP-A2 CTxB>Cer and BL21(DE3) pET30a<MBP-A2 CTxB>Cer variants extracellular expression of MBP-A2 and digested MBP. (**b**) V2 pET30a T70.09<MBP-A2 CTxB>Cer and BL21(DE3) pTRBAB5-G1S <MBP-A2 CTxB>Cer variants extracellular expression of MBP-A2 and digested MBP. (**c**) Enzymatically lysed 1mg CDM of V1 pET30a<MBP-A2 CTxB>Cer, BL21(DE3) pET30a<MBP-A2 CTxB>Cer, V2 pET30a T70.09<MBP-A2 CTxB>Cer and BL21(DE3) pTRBAB5-G1S <MBP-A2 CTxB>Cer variants shows an intracellular expression of MBP-A2 and digested MBP. CS-Cell soluble and IBs-Inclusion Bodies.

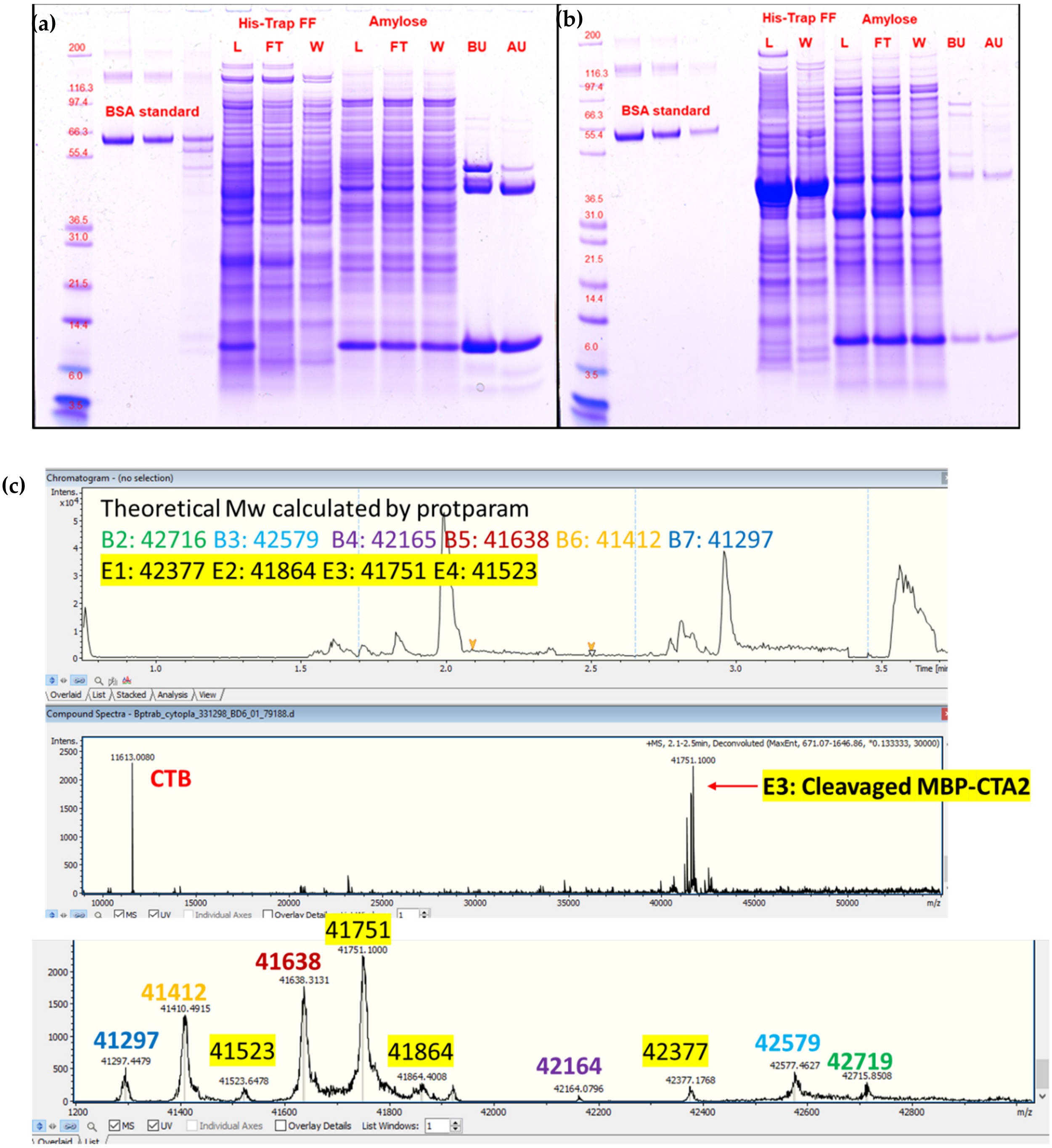

Figure S8. Homogenized 10g Wet Cell Mass (WCM), purified with IMAC and Amylose column. SDS-PAGE contains (**a**) V1 pET30a T70.09 <MBP-A2 CTxB>Cer and (**b**) BL21 (DE3) pTRBAB5-G1S <MBP-A2 CTxB>. L-loading, FT-flow through, W-wash, BU-before ultrafiltration, and AU-after ultrafiltration. (**c**) MS analysis of purified cell homogenate (AU) with MBP-A2 peaks cleaved at the different positions after purification. Cut sites are located on a linker between MBP and A2 helix.

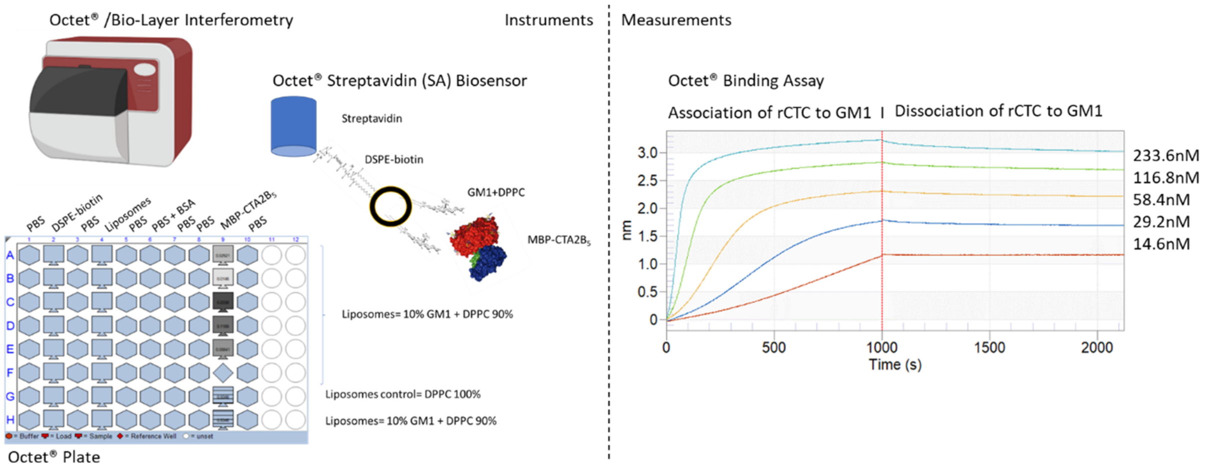

Figure S9. BLI analysis of rCTC and GM1 liposome binding.

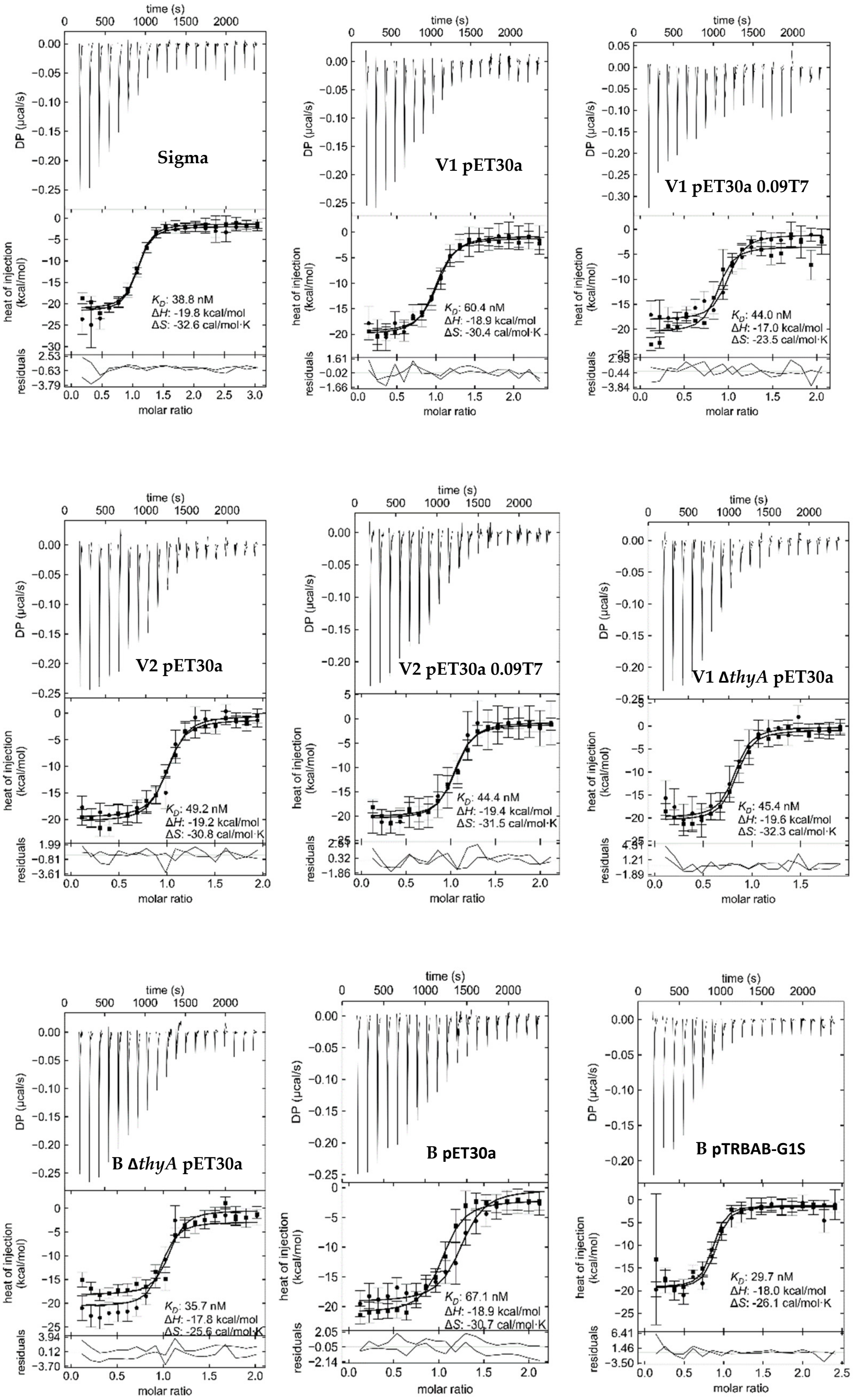

Figure S10. Thermograms and binding isotherms of all variants.

Table S1. Thermodynamic parameters for rCTC: GM1 binding interaction using ITC.

Scheme 10.	Repeat[a]	Binding site conc[b] (μM)	Correction factor for binding site conc[c]	Best-fit ΔH kcal/mol	Low range[d] (ΔH)	High range[d] (ΔH)	Best-fit $Log_{10}[K_{AB}]$[e]	Low range $Log_{10}[K_{AB}]$[d]	High range $Log_{10}[K_{AB}]$[d]
**Classic CTxB (Sigma)**	1	3.5	1.038	-19.83	-21.03	-18.75	7.41	7.21	7.63
	2		1.046						
**C41 MBP-CTA2B5[f]**	1	4.6	1.096	-17.99	-20.39	-16.07	7.40	6.84	8.14
	2		1.086						
**V1 pET30a**	1	4.5	1.075	-18.90	-20.21	-17.77	7.22	6.99	7.47
	2		1.043						
**V1 0.09T7 pET30a**	1	5.1	1.034	-17.04	-20.08	-14.82	7.36	6.80	8.21
	2		0.909						
**V2 pET30a**	1	5.4	0.980	-19.17	-20.78	-17.75	7.31	7.01	7.64
	2		0.957						
**V2 0.09T7 pET30a**	1	4.9	1.079	-19.43	-20.80	-18.22	7.35	7.07	7.68
	2		1.099						
**V1 *ΔthyA* pET30a**	1	5.5	0.998	-19.64	-21.25	-18.20	7.34	7.07	7.65
	2		1.016						
**B *ΔthyA* pET30a**	1	5.2	1.032	-17.78	-20.32	-15.72	7.45	6.95	8.22
	2		0.949						
**B pET30a**	1	4.9	1.213	-18.94	-20.85	-17.37	7.17	6.87	7.51
	2		1.010						
**B pTRBAB5-G1S**	1	4.3	1.034	-18.04	-19.87	-16.44	7.34	7.15	7.99
	2		0.989						

[a]ITC experiments were performed in duplicate; [b]binding site concentrations are 5 × rCTC AB_5 concentration; [c]separate correction factors were allowed for each of the two samples during the global fitting of the data; [d]High and low range values are based on a 68.3% confidence interval; [e]fitting in SEDFIT is based on $Log_{10}[K_{AB}]$, i.e., log of the association constant K_a. Values for K_d for each sample are shown in Figure S11; [f]Sample expressed from pTRBAB-G1S in *E. coli* C41 cells.

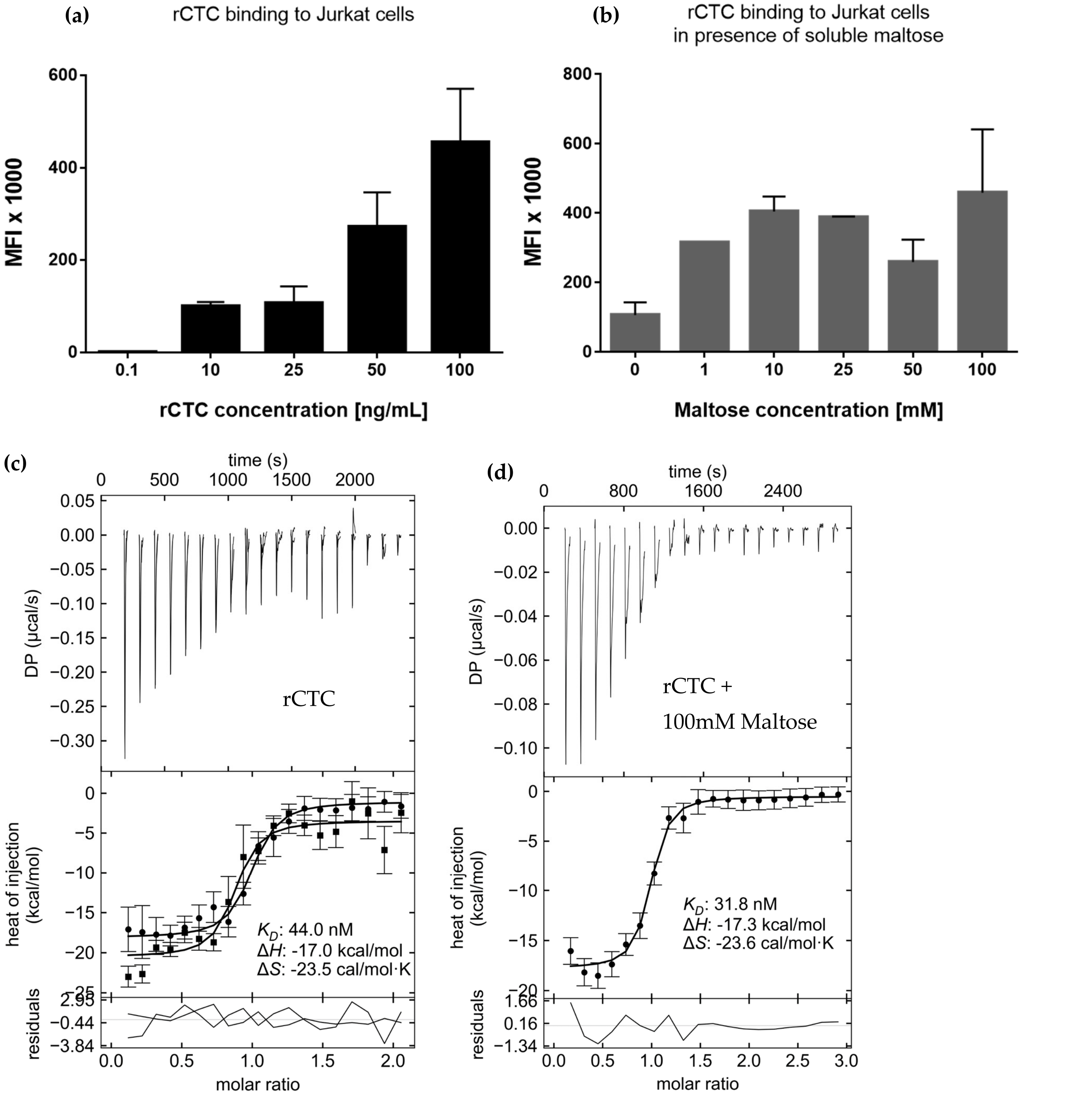

Figure S11. Binding studies of rCTC to Jurkat cells in presence of soluble maltose. (**a**) Quantification of flow cytometry analysis of gated living Jurkat cells incubated for 30 min at 4 °C with increasing concentrations of rCTC (93 pM, 0.23 nM, 0.46 nM, 0.93 nM). (**b**) Quantification of flow cytometry analysis of Jurkat cells stimulated with 0.23 nM rCTC pre-incubated with increasing concentrations of soluble maltose for 30 min at RT (rCTC 0.23 nM, rCTC 0.23 nM + 1 mM maltose, rCTC 0.23 nM + 10 mM maltose, rCTC 0.23 nM + 25 mM maltose, rCTC 0.23 nM + 50 mM maltose, rCTC 0.23 nM + 100 mM maltose). The 1, 10, 25, 50 and 100mM maltose concentrations were used to saturate MBP-binding sites. The flow cytometry analysis revealed an increase in fluorescence intensity when rCTC is treated with soluble maltose. (**c**) ITC interaction measurement between 3.6µM rCTC (PBS) and 50µM GM1. (**d**) ITC interaction measurment between 3.6 µM rCTC (PBS) and 50µM GM1 and in precence of 100mM maltose. There is no significant change of the binding affinity between rCTC and GM1 with/without 100mM Maltose.

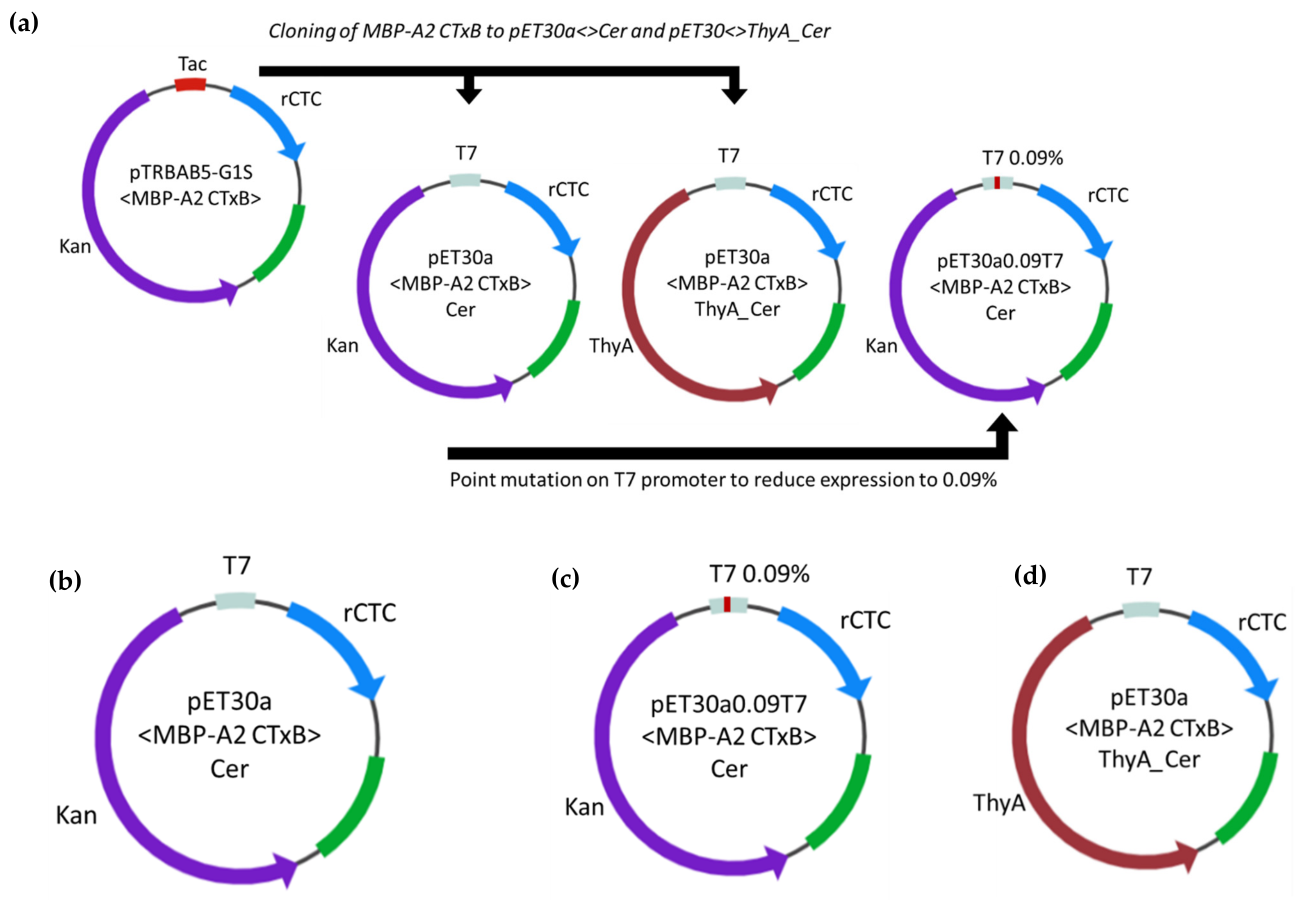

BsaI cloning:

The pTRBAB5-G1S insert:

Primer1 (27-mer):
AGGTCTCCATGAAAATAAAAACAGGTGC

Primer2 (28-mer):
AGGTCTCCTTAGTTTGCCATACTAATTGC

The pET30a<>Cer vector:

Primer3 (27-mer):
AGGTCTCCCTAATAAATTCGAACGCCAG

Primer4 (34-mer):
AGGTCTCCTCATATGTATATCTCCTTCTTAA
AGTT

Blunt end cloning:

Primer6 (22-mer):
GGGGAATTGTGAGCGGATAAC
A

Primer7 (33-mer):
TATAGTGAGTCG-
TATGAATTTCGCGG-
GATCGAG

BsaI cloning:

The ThyA insert
(‘in house’ pET28a<IL-8 ThyA>Cer)

Primer8 (26-mer):
AGGTCTCTATTTCACAGCAAACAC
CAC

Primer9(24-mer):
AGGTCTCTTTGGGGAACTGCCGAC
T

The pET30a<MBP-A2 CTxB>Cer vector:

Primer10(25-mer):
AGGTCTCTCCAAAATCCCTTAACG
TG

Primer11 (35-mer):
AGGTCTCTAAATTGTAAAC-
GTTAATATTTTGTTAAA

Figure S12. Cloning of all construct generated for expression of rCTC. (**a**) A general outline of cloning was performed. (**b**) BsaI (restriction enzyme II) cloning of pET30a<MBP-A2 CTxB>Cer construct. (**c**) Blunt end cloning of pET30a 0.09T7<MBP-A2 CTxB>Cer construct. (**d**) BsaI cloning of pET30a<MBP-A2 CTxB>ThyA_Cer construct.

Table S2. Primers selected for qPCR analysis.

Gene of interest	Primers	Efficiency [%]
cysG	GGTTGCTGTTAGACGCAGGC<br>CATCTGCCCATGCGGTGAAC	**111**
rssA	GCTGGTTGATGGAGCAGTCG<br>TGCTGCAGGTCAACCGCTAT	**105**
gspD	GATGCGGGTAGCGTCGGTAA<br>TCCGCTGACGCATATTCCAGA	**106**
gspE	CGTATCCACACTGCCGTCCA<br>GCGTGGTGCTTTTACCGGAG	**111**
gspG	TATCAAGCGTCTGCCTGCCG<br>GATGTCGTCCTCGGTTCCCA	**104**
mbp-a2	TCCGCTTTCTGGTATGCCGT<br>AATCCTTCCCTCGATCCCGA	**93**
ctxB	TCACGAGCAATTGACCAACAAGG<br>TGCATGCGCCTGAACAGATAC	**95**
slyA	TACGGACCAGTGATGGCTGC<br>TCTGATCTGGCACGGTTGGT	**109**
fur	CGATAACCTTGCCGCAGTCG<br>GGTATCGTCACCCGCCACAA	**95**

Table S3. Content of Media and Trance Element solution.

Trace Elements solution	Weight dissolved in 5M HCl
$FeSO_4.7H_2O$ [g/L]	40
$MnSO_4 .H_2O$ [g/L]	10
$AlCl_3.6H_2O$ [g/L]	10
$CoCl_2$ $6H_2O$ [g/lL	7.3
$ZnSO_4.7H_2O$ [g/L]	2
Na_2MoO_4 $2H_2O$ [g/L]	2
$CuCl_2.2H_2O$ [g/L]	1
H_3BO_3 [g/L]	0.5
**Batch Media (2.4g CDM)**	**Weight/g CDM**
KH_2PO_4 [g]	0.09
85% H_3PO_4 [g]	0.03
Yeast Extract [g]	0.15
Na-Citrate dihydrate [g]	0.04
Mg-Chloride 6xH_2O [g]	0.05
Ca-Chloride 2xH_2O [g]	0.02
Trace element solution [µL]	50.00
Ammonsulfate [g]*	0.05
Glucose*	7.27
fill up to with H_2O [g]	600
**Fed-batch Media (53.6g***)**	**Weight/g CDM**
KH_2PO_4 [g]	0.09
85% H_3PO_4 [g]	0.03
Na-Citrate dihydrate [g]	0.04
Mg-Chloride 6xH_2O [g]	0.05
Ca-Chloride 2xH_2O [g]	0.02
Trace element solution [ml]	0.05
Glucose*	179
fill up to with H_2O [g]	550

* Calculated for final CDM (56g). ** Glucose coefficient = 0.33. *** Feed prepared including 1.1 safety factor.
